# Efficacy and safety of Zhibitai in combination with atorvastatin for lipid lowering in patients with coronary heart disease

**DOI:** 10.18632/oncotarget.18329

**Published:** 2017-06-01

**Authors:** Danyan Xu, Jiahui Hu, Qinghua Wu, Zhiming Du, Yusheng Xue, Xu Zhang, Yi Li, Yushan Chen, Xiaoping Chen, Hong Zhang, Shuiping Zhao

**Affiliations:** ^1^ Department of Cardiology, the Second Xiangya Hospital of Central South University, Changsha, P.R. China; ^2^ Department of Cardiology, the Second Affiliated Hospital of Nanchang University, Nanchang, P.R. China; ^3^ Department of Cardiology, the First Affiliated Hospital of Sun Yat-sen University, Guangzhou, P.R. China; ^4^ Department of Cardiology, Tangdu Hospital, Xi’an, P.R. China; ^5^ Department of Chinese Medicine, Huadong Hospital Affiliated to Fudan University, Shanghai, P.R. China; ^6^ Department of Chinese Medicine, Beijing Hospital, Beijing, P.R. China; ^7^ Center of Cardiology, the First Affiliated Hospital of Henan University of TCM, Zhengzhou, P.R. China; ^8^ Department of Cardiology, West China Hospital of Sichuan University, Chengdu, P.R. China; ^9^ Department of Cardiology, the First People’s Hospital of Yunnan, Kunming, P.R. China; ^10^ Department of Cardiology, the Second Xiangya Hospital of Central South University, Changsha, P.R. China

**Keywords:** Zhibitai, atorvastatin, combination therapy, dyslipidemia, atherosclerosis

## Abstract

**Background:**

Zhibitai, a natural lipid-lowering Chinese medicine, is well tolerated in patients and has low incidence of adverse events. In this study, we evaluated the efficacy, safety, and side effects of Zhibitai in combination with low dose Atorvastatin compared to high dose Atorvastatin in patients with coronary heart disease or at high risk of coronary heart disease.

**Methods:**

This was a randomized, double-blind, multi-center clinical trial on 720 patients with coronary heart disease or at high risk of coronary heart disease. The patients were randomly assigned to a Zhibitai-Atorvastatin group (480 mg Zhibitai twice daily plus 10 mg atorvastatin once daily) or Monotherapy group (40 mg Atorvastatin once daily). Blood samples were obtained at baseline, week 4, and week 8 after a minimum 8-hour fast. Efficacy was evaluated in terms of the changes in the following parameters: lipoprotein profiles [total cholesterol (TC), triglyceride (TG), low-density lipoprotein cholesterol (LDL-C), high-density lipoprotein cholesterol (HDL-C)]. Safety was assessed throughout the study by clinical laboratory tests including liver function [alanine transaminase, aspartate transaminase] and renal function [blood urea nitrogen], and creatine kinase; physical examination; and adverse events monitoring.

**Results:**

TC, TG, LDL-C levels were significantly decreased andHDL-C levels were significantly increased at week 4 and week 8 (all *P* < 0.05) in both groups but had no significant differences between the two groups (*P* > 0.05). In subgroup analyses, Zhibitai-Atorvastatin Group produced significantly greater reduction in TG compared with Monotherapy Group at week 8 in patients with TG > 203.72mg/dL (*P* < 0.01). Among patients with LDL-C levels > 131.48 mg/dL, Zhibitai-Atorvastatin Group produced a greater reduction of LDL-C levels compared with the Monotherapy Group at week 4 (*P* < 0.05). The incidence of liver dysfunction, headache, or gastrointestinal intolerance was significantly lower in the Zhibitai-Atorvastatin Group compared with Monotherapy Group during the 8-week study peroid (*P* < 0.001). There were no significant differences in renal function, myopathy, and other adverse events between the groups.

**Conclusion:**

Overall the two groups have similar lipid regulation efficacy. Zhibitai plus low dose Atorvastatin is more efficacious in lowering TG in patients with TG > 203.72 mg/dL at week 8. There are fewer side effects in Zhibitai plus low dose Atorvastatin group. Long term follow up is required to evaluate cardiovascular outcomes.

## INTRODUCTION

Low-density lipoprotein cholesterol (LDL-C) reduction remains the cornerstone of atherosclerotic cardiovascular disease (ASCVD) prevention [1–2]. Statin is the primary treatment for reducing ASCVD risk for patients in whom lipid-lowering therapy is indicated [6–7]. European and American National Lipid Association guidelines recommend lowering LDL-C to < 69.60 mg/dL or by 50% in patients with established ASCVD [3–5]. It usually requires moderate or even intensive statin to reach the target levels [8]. A range of sources support a dose relation for statin adverse events and dropouts [9–12]. Accordingly, combination low dose statin with different lipid-lowering agents to reach the recommended target levels and to reduce dose related adverse events is recommended [13].

Based on the collective evidence from several large cardiovascular outcome trials, the combination therapy with the use of Niacin [14] or Fibrates and statin didn’t significantly reduce the risk of cardiovascular events but might increase serious adverse events, as compared with statin alone [15–17]. The natural lipid-lowering drugs made from Chinese Herb have fewer adverse events and are well tolerated, and would become potential alternative drugs in combination with statins in ASCVD prevention [18].

The *Di’ao Zhibitai* compound capsule (Zhibitai), a natural lipid-lowering Chinese medicine, is comprised of four key ingredients, hawthorn, atractylodes rhizome, alismatis rhizoma, and, *Monascus* spp. (also termed Red yeast rice extract). The major component of Zhibitai is a natural plant lovastatin acid, 3-hydroxy-3-methylglutaryl coenzyme A (HMG-CoA) reductase. Hawthorn aids digestion, regulates blood lipoprotein, improves antioxidant, and regulates immune function. Atractylodes rhizome decreases LDL-C, increases HDL-C, and decreases anti-platelet aggregation. These components have impressive synergistic effects with different mechanisms in regulating lipoprotein levels [19]. A number of small studies have evaluated the efficacy and safety of Zhibitai compared with Atorvastatin in patients with coronary heart disease (CHD) or at high risk for CHD [20–21, 23]. Our team has demonstrated that intensive Zhibitai therapy is as effective as Atrovastatin in lowering LDL-C, and presumably in reducing the risk of CV events [21]. Our data has also shown that *Xuezhikang* (a Chinese patent medicine with some same major ingredients of Zhibitai) plus low dose Rosuvastatin is more effective in lowering LDL-C levels and safer compared with high dose Rosuvastatin alone [22].

Accordingly, we propose that combined use of Zhibitai and statin is superior to statin monotherapy. Ans this study is to compare the efficacy and safety of the combination of Zhibitai and low dose Atorvastatin with high dose Atorvastatin alone in patients with CHD or at high risk of CHD.

## RESULTS

### Patients

A total of 720 patients were enrolled in the study, 10 of them were excluded due to lack of clinical laboratory lipoprotein profiles (4 patients from the Zhibitai-Atorvastatin Group, 6 patients from the Monotherapy Group) before drug administration. 353 subjects were assigned into the Zhibitai-Atorvastatin Group and 357 into the Monotherapy Group. Another 14patients were excluded after the run-in phase (3 patients from the Zhibitai-Atorvastatin Group; 11 patients from the Monotherapy Group) due to poor compliance or lack of information (Table 1). The difference of the drop-out/elimination rate between the Zhibitai-Atorvastatin Group (1.96%) and the Monotherapy Group (4.68%) was significant (*P* = 0.0419), and the major reason for dropout in the Monotherapy group was poor compliance as noted in Table 1. Both groups were well matched in age, sex, height, body mass index (BMI), systolic blood pressure (SBP), diastolic blood pressure (DBP), smoking status, and diabetes (Table 2).

**Table 1:** List of excluded patients

Group	Total( male/female)	Reasons
Zhibitai-Atorvastatin Group	4(3/1)	Lacking clinical laboratory lipid profiles
Zhibitai-Atorvastatin Group	3(1/2)	Poor compliance
Monotherapy Group	6(3/3)	Lacking information of lipoprotein
Monotherapy Group	11(8/3)	Poor compliance

**Table 2:** Baseline characteristics of the patients (FAS)

Characteristics	Zhibitai-Atorvastatin Group (*n* = 353)	Monotherapy Group (*n* = 357)
Age, years	58.31±9.82	57.20±9.84
Sex (male/female), no.	157/196	147/210
Height, cm	164.81±7.72	165.82±8.09
Weight, kg	66.84±10.54	67.66±10.66
BMI, kg/cm^2^	24.55±3.04	24.52±2.86
SBP, mmHg	135.10±17.20	134.65±16.97
DBP, mmHg	82.53±11.16	81.41±10.56
Smokers, no. (%)	130(36.83)	133(37.25)
Patients with CHD or at high risk of CHD, no. (%)	233(66.01)	241(67.51)

BMI: body mass index; SBP: systolic blood pressure; DBP: diastolic blood pressure

### Lipid profile

#### Changes in lipid profile

TC, TG, and LDL-C levels were decreased significantly at week 4 and week 8 compared with baseline in the two groups (*P* < 0.001); HDL-C levels were significantly increased compared with baseline in the two groups (*P* < 0.001). There was no significant difference between the two groups at baseline, week 4, and week 8 (*P* > 0.05) (Table 3).

**Table 3:** Changes in lipoprotein profiles in the two groups

Group/Index	Zhibitai-Atorvastatin Group (*n* = 353)			Monotherapy Group (*n* = 357)		
	Week 0	Week 4	Week 8	Week 0	Week 4	Week 8
TC (mg/dL)	216.55±51.43	186.39±43.31^a^	170.53±40.99 ^b^	217.71±56.46	184.45±45.63^a^	169.76±41.76^b^
TG (mg/dL)	232.06±134.63	186.89±104.51^a^	157.66±71.74 ^b^	232.95±143.49	188.66±113.37^a^	168.29±101.86 ^b^
LDL-C(mg/dL)	128.38±41.76	106.34±35.58 ^a^	95.24±34.48 ^b^	133.02±43.70	107.89±37.90 ^a^	96.64±34.42 ^b^
HDL-C(mg/dL)	45.63±17.40	48.72±18.17^a^	51.82±16.63 ^b^	43.70±13.15	46.79±15.85 ^a^	49.50±15.08 ^b^

Compared with week 0, ^a^*P* < 0.001, ^b^*P* < 0.0001.

TC: total cholesterol; TG: triglyceride; LDL-C: low-density lipoprotein cholesterol; HDL-C: high-density lipoprotein cholesterol

#### Changes in lipid profile in patients with TG > 203.72 mg/dL

Among patients with TG > 203.72 mg/dL, TG levels were significantly lower in the Zhibitai-Atorvastatin Group than in the Monotherapy Group at week 8 (*P* < 0.01). Compared with baseline, TC and LDL-C levels were significantly decreased and HDL-C levels were significantly increased at week 4 and week 8 in both groups (*P* < 0.05). There was no difference between the two groups in the change of TC levels, LDL-C and HDL-C levels (*P* > 0.01) (Table 4).

**Table 4:** Changes in lipoprotein profiles in patients with TG >203.72mg/dL

Group/Index	Zhibitai-Atorvastatin Group(*n*=181)			Monotherapy Group(*n*=175)		
	Week 0	Week 4	Week 8	Week 0	Week 4	Week 8
TC (mg/dL)	226.60±52.59	192.58±42.92^b^	174.01±39.44^b^	235.89±55.29	196.06±48.72^c^	179.25±42.54^c^
TG (mg/dL)	317.09±137.29	230.23±106.29^b^	186.01±80.60^a,b^	325.07±154.12	250.67±130.20^c^	216.12±122.23^c^
LDL-C(mg/dL)	130.70±41.76	105.18±34.42^b^	93.58±32.10^b^	141.53±44.86	113.69±40.60^c^	99.77±36.74^c^
HDL-C(mg/d)	44.08±15.85	48.72±19.72^b^	51.82±15.47^b^	42.54±10.83	46.79±11.99^c^	49.88±15.47^c^

Compared to Monotherapy Group,^a^*P* < 0.01; Compared to Zhibitai-Atrovastatin Group at week 0, ^b^*P*<0.05; Compared to Monotherapy Group at week 0, ^c^*P*<0.05.

TC: total cholesterol; TG: triglyceride; LDL-C: low-density lipoprotein cholesterol; HDL-C: high-density lipoprotein cholesterol.

#### Changes in lipid profile in patients with LDL-C > 131.48 mg/dL

Among the patients with LDL-C > 3.4 mmol/L, LDL-C, TG, and TC levels were significantly decreased, and HDL-C was significantly increased at week 4 and week 8 compared to baseline in both groups. The LDL-C was significantly lower in Zhibitai-atorvastatin Group than in the Monotherapy Group at week 4. However, the difference of LDL-C between the two groups was not significant at week 8 (*P* > 0.05). There was no difference of HDL-C, TG, and TC in the two groups at week 4 and week 8 (*P* > 0.05) (Table 5).

**Table 5:** Changes in lipid profiles in patients with LDL-C > 131.48mg/dL

Group/Index	Zhibitai-Atorvastatin Group (*n* = 172)			Monotherapy Group (*n* = 170)		
	Week 0	Week 4	Week 8	Week 0	Week 4	Week 8
TC(mg/dL)	243.23±36.74	199.15±40.99^b^	182.91±41.76^c^	250.58±45.63	206.88±44.08^b^	187.16±41.38^c^
TG(mg/dL)	231.18±132.86	185.12±103.63^b^	155.00±66.43^c^	246.24±138.18	200.18±118.69^b^	170.95±85.92^c^
LDL-C(mg/dL)	162.03±25.14	122.20±34.03^a, b^	108.28±33.64^c^	168.21±30.94	129.93±36.74^b^	113.69±34.80^c^
HDL-C(mg/dL)	46.84±14.31	48.72±12.76^b^	52.59±15.08^c^	45.63±12.76	49.88±18.95^b^	53.36±17.03^c^

Compared to Monotherapy Group at week 4,^a^*P* < 0.05; Compared to Zhibitai-Atrovastatin Group at week 0, ^b^*P*<0.05; Compared to Monotherapy Group at week 0, ^c^*P*<0.05.

TC: total cholesterol; TG: triglyceride; LDL-C: low-density lipoprotein cholesterol; HDL-C: high-density lipoprotein cholesterol.

### Safety

#### Clinical laboratory tests

There were no elevations of creatine kinase more than 10 times the upper limit of normal range at any time. Elevations of ALT were 4.61% and 3.22% at week 4 and week 8 respectively in the Zhibitai-Atorvastatin group and 6.98% and 10.38% at week 4 and week 8 respectively in the Monotherapy group. Elevations of AST were 3.60% and 1.90% at week 4 and week 8 respectively in the Zhibitai-Atorvastatin group and 4.71% and 8.28% at week 4 and week 8 respectively in the Monotherapy group. There were no significant differences in renal function and CK between the two groups (*P* > 0.05) (Table 6). Elevations of ALT and AST were lower in Zhibitai-Atorvastation Group than Atorvastatin Group both in subgroup TG > 203.72 mg/dL and subgroup LDL-C > 131.48mg/dL with the same results as the entire study population. (Supplementary Table 1 and Supplementary Table 2).

**Table 6:** Occurrence of abnormal clinical laboratory tests [no. (%)]

Group/Index	Zhibitai-Atorvastatin Group (*n* = 353)			Monotherapy Group (*n* = 357)		
	Week 0	Week 4	Week 8	Week 0	Week 4	Week 8
ALT(abnormal)	0(0.00)	14(4.61)	10(3.22) ^a^	0(0.00)	21(6.98)	30(10.38)^a^
AST(abnormal)	0(0.00)	11(3.60)	6(1.90) ^b^	0(0.00)	14(4.71)	24(8.28)^b^
BUN(abnormal)	0(0.00)	9(3.38)	9(3.20)	0(0.00)	7(2.31)	15(5.08)
Creatine(abnormal)	0(0.00)	6(2.21)	7(2.46)	0(0.00)	6(1.96)	9(3.00)
CK(abnormal)	0(0.00)	5(2.39)	9(4.37)	0(0.00)	5(2.42)	7(3.52)

Compared with Monotherapy Group at week 8, ^a^*P* < 0.001, ^b^*P* < 0.001. ALT: Alanine transaminase (the definition of ALT abnormal was >3 times the upper limit of normal range); AST: aspartate transaminase (the definition of ALT abnormal was >3 times the upper limit of normal

range); BUN: blood urea nitrogen (the definition of BUN abnormal was > the upper limit of normal range); CK: creatine kinase (the definition

of ALT abnormal was>10 times the upper limit of normal range).

### Adverse events

There were no serious adverse events and adverse events were rare. The incidence of gastrointestinal intolerance was 0.28% *vs*. 7.63% at week 4 and 0.28% *vs*. 7.39% at week 8 in the two groups as noted in Table 7. The incidence of headache was 0.85% *vs*. 3.47 at week 4 and 0.57% *vs*. 2.60% at week 8 in the two groupd respectively) (*P* < 0.05). There was no difference of incidence of insomnia, depression, dyspepsia, and other adverse events in the two groups. There was some difference in the incidence of stomachache and myalgia at baseline and at week 4 and week 8, but the changes have no significant differences (all, *P* > 0.05) (Table 7).

**Table 7:** Occurrence of side effects [no. (%)]

Group/Side effects	Zhibitai-Atorvastatin Group			Monotherapy Group		
	Week 0 (*n*=353)	Week 4 (*n*=353)	Week 8 (*n*=351)	Week 0 (*n*=357)	Week 4 (*n*=354)	Week 8 (*n*=352)
Insomnia	30(8.5%)	22(6.23%)	12(4.27%)	21(5.88%)	19(5.73%)	16(4.55%)
Depression	6(1.7%)	1(0.28%)	0(0.00%)	5(1.41%)	5(1.41%)	3(0.85%)
Dyspepsia	10(2.83%)	7(1.98%)	6(1.71%)	14(3.92%)	14(3.95%)	12(3.41%)
Diarrhea	2(0.57%)	0(0.00%)	0(0.00%)^a^	4(1.12%)	7(1.98%)	7(1.99%)
Nausea	4(1.13%)	1(0.28%)	1(0.28%)^a^	8(2.24%)	20(5.65%)	19(5.40%)
Headache	7(1.98%)	3(0.85%)	2(0.57%)^a^	12(3.36%)	12(3.47%)	9(2.60%)
Coelialgia	1(0.28%)	0(0.00%)	3(0.86%)^a^	10(2.80%)	11(3.17%)	8(2.31%)
Myalgia	0(0.00%)	0(0.00%)	0(0.00%)^a^	8(2.24%)	10(2.89%)	8(2.31%)
Others	2(0.57%)	3(0.85%)	2(0.57%)	0(0.00%)	1(0.28%)	0(0.00%)

Compared with Monotherapy Group at week 8, ^a^*P* < 0.001

## DISCUSSION

TC, TG, and LDL-C levels were significantly decreased and HDL-C levels were significantly increased at 4 and 8 weeks in the Zhibutai-Atorvastatin Group and Monotherapy Group. In subgroup analyses, TG levels were significantly lower at week 8 in the Zhibitai-Atorvastatin Group than in the Monotherapy Group among patients with TG > 203.72 mg/dL, and LDL-C levels were significantly lower at week 4 in the Zhibitai-Atorvastatin Group than in the Monotherapy Group among patients with LDL-C > 131.48 mg/dL.

Our group previously has demonstrated that compared with 10mg atorvastatin, 480mg Zhibitai (twice daily), equal dosage of this research, could also decrease LDL-C, TG, TC level after 8 weeks of treatment, and the difference between these two groups had no statistically significance [22]. Further research of Xiang [24] also have found that 480mg Zhibitai (twice daily) was equal to 10mg atorvastatin in lowering TC, TG and LDL-C level, and rising HDL-C level. This research and ours reflected that 480mg Zhibitai twice daily had equal effect in regulating lipoprotein levels compared to 10mg atorvastatin.

It is well known that statins could increase protein convertase subtilisin/kexin type 9 (PCSK9) protein levels, which binds the hepatic LDL-C receptor (LDLR), causing LDLR subsequent degradation and then increasing circulating LDL-C level [25–29]. So increasing dose of statins failed to achieve propotional LDL-C lowering and the descend range of LDL-C only increased 5%-6% with the dosage doubling.

In subgroup of LDL-C > 131.48mg/dL, Zhibitai-Atorvastatin Group showed advantage in lowering LDL-C levels comparing with Monotherapy at week 4. So our initial idea was that better effect of Zhibitai in lowering LDL-C level might be due to Zhibitai-induced decreasing in PCSK9 levels.

First, we have proved that statin could up-regulate the expression of PCSK9 level with dose-independent manner (Supplementary Figure 1). Then, Zhibitai was used to challenge starving HepG2 cells, but what made us surprised was that PCSK9 was also increased with the increasing dosage of Zhibitai (Supplementary Figure 2), which means the better efficacy of Zhibitai-Atorvastatin therapy in lowering LDL-C level didn’t rely on decreasing PCSK9.

Then whether there exists another pathway of Zhibitai in lowering LDL-C level. The sterol-responsive nuclear liver X receptor (LXR) helps maintain cholesterol homeostasis, not only through promotion of cholesterol efflux but also through suppression of low-density lipoprotein (LDL) uptake. LXR inhibits the LDL receptor (LDLR) pathway through transcriptional induction of Idol (inducible degrader of the LDLR), an E3 ubiquitin ligase that triggers ubiquitination of the LDLR on its cytoplasmic domain, thereby targeting it for degradation [39]. So in our following research, we detected the expression of LXR, a specific element in the upstream related to Idol. However, it was shown no down-regulation of LXR with increasing dosage of Zhibitai (Supplementary Figure 3), means that the target of Zhibitai was not LXR-Idol-LDLR axis either

The Chinese medicine *Zhibitai* capsule is comprised of monacolins, hawthorn, alismatis, and atractylodes rhizome. The four components in Zhibitai have synergistic effect in regulating lipoprotein levels and the mechanism of Zhibitai remains to be further studied [30–32].

Previous research by our group proved that 480 mg Zhibitai therapy significantly decreases plasma TG at week 4, while 10 mg atorvastatin only decreases TG at week 8. From this small-scale study, Zhibitai-Atrovastatin can reduce TG levels more than high dose Atrovastin among patients with TG > 203.72 mg/dL at week 8, which indicates Zhibitai may be more effective in reducing TG than Atorvastatin. In addition to the natural statin, monacolins, Zhibitai also contains linoleic acid (which comprises 48.13% of the total fatty acids in Zhibitai), which might contribute to reducing TG levels [33–34] .The benefit of lowering TG would provide an option to Asian patients who mainly have hypertriglyceridemia and who favor traditional Chinese medicine.

Our study also shows that Zhibitai-atorvastatin is better at lowering LDL-C levels compared with Atorvastatin alone in patients with LDL-C > 131.48 mg/dL mmol/L at week 4. The hawthorn component in Zhibitai contains ursolic acid and oleanolic acid, which can also decrease LDL-C [35]. Alismatis can decrease TC, TG, and LDL-C levels by reducing fat absorption from the intestine and cholesterol synthesis in the liver, and promote insulin release and decreases glucose levels [36]. These components have a synergistic effect on regulating lipoprotein levels [19].

There were fewer adverse events in the Zhibitai-Atorvastatin Group and lower incidence of liver dysfunction compared with high dose of Atorvastatin. Gastrointestinal intolerance is less in the Zhibitai-Atorvastatin group. The hawthorn in Zhibitai capsules aids digestion and has a positive effect on blood lipid profiles. Zhibitai capsules are comprised of common Chinese traditional medicines that are safely manufactured and approved by China Food and Drug Administration. All components in the Zhibitai capsules are commonly used, safe, and non-toxic and are widely used in some food preparations. In the present study, the incidence of adverse events in the the Zhibitai-Atrovastatin group was lower. The Zhibitai capsules were well tolerated and easily accepted by the patients with a better compliance.

The follow-up period of this study is only up to 8 weeks, which is not long enough to show the cardiovascular outcomes. Long term follow up will be required to provide the evidence of the combination therapy with the use of Zhibitai and low-dose Atorvastatin in the reduction of the risk of cardiovascular events.

The LDL-C, TC, and TG levels in both groups were decreased effectively. However, the combination of Zhibitai and Atorvastatin was more effective in lowering TG and LDL-C levels in patients with hypertriglyceridemia and with LDL-C > 131.48 mg/dL mmol/L. Zhibitai plus atorvastatin was effective, safe, and well tolerated.

## MATERIALS AND METHODS

### Study patients

A total of 720 patients (310 men and 410 women) aged 25-89 years with CHD or at high risk of CHD were enrolled from 11 medical centers between 2011 and 2012.

The diagnosis of CHD or at high risk of CHD was made according to the Chinese guidelines on the prevention and treatment of dyslipidemia in adults as follows: (1) CHD was defined as acute coronary disease, including unstable angina (UA), acute myocardial infarction (AMI), stable angina pectoris, old MI, myocardial ischemia with objective evidence, and following percutaneous coronary intervention or coronary artery surgery; (2) ischemic stroke, peripheral arterial disease, abdominal aortic aneurysm, symptomatic carotid artery disease, and diabetes, multiple risk factors (hypertension, smoking, obesity, male ≥ 45 years old or female ≥ 55 years old with first-degree relative with CV diseases, or male ≥ 55 years old or female ≥ 65 years old); and (3) major adverse coronary events equivalent to CHD. [13, 37–38]

The subjects must fulfill one of the following inclusion criteria to be eligible for participation: (1) TC > 200.31mg/dL or LDL-C > 130.32mg/dL; (2) TG level > 150.58mg/dL; (3) HDL-C < 40.22mg/dL.

The exclusion criteria were: (1) MI within 3 months of study recruitment, cerebrovascular accidents within 6 months, severe trauma or major surgery, nephritic syndrome (NE), hypothyreosis, acute or chronic hepatobiliary disease and familial hypercholesterolemia; (2) hyperlipidemia caused by drugs such as phenothiazine, adrenergic beta blockers, cortical hormones, and acyeterion; (3) taking heparin, anti-thyroid drugs, or other drugs that would affect serum lipid metabolism; (4) pregnant or nursing women, statin and Zhibitai allergy, or psychosis; (6) poor cardiac function, i.e., grade IV according to the New York Heart Association (NYHA).

### Research methodology

#### Study design

This was a randomized, controlled, double-blind, multi-center clinical trial. 720 patients were randomly assigned in a 1:1 ratio into the Zhibitai-Atorvastatin Group (*n* = 357, age 58.31 ± 9.82 years, 160 men and 197 women) or the Monotherapy Group (*n* = 363, age 57.69 ± 9.87 years, 150 men and 213 women). During the 8 week study period, patients in the Zhibitai-Atorvastatin Group received 480 mg of Zhibitai (manufactured by Chendu Di’ao Pharmaceutical Group Co., Ltd.) twice daily and 10 mg of Atorvastatin once daily; patients in the Monotherapy Group received 40 mg of Atorvastatin once daily. The research ethics committee of Central South University approved the study. Each patient provided written informed consent.

### Efficacy and safety assessment

During the double-blind period, the efficacy was evaluated by measuring TC, TG, HDL-C, and LDL-C levels at baseline, week 4, and week 8 respectively. Safety was assessed by clinical laboratory tests including alanine transaminase (ALT), aspartate transaminase (AST), creatine, blood urea nitrogen (BUN), creatine kinase (CK); physical examination; and adverse events monitoring. Blood samples were collected after a minimum 8-hour fast.

### Statistical analysis

All statistical analyses were performed using SAS software (version 9.13) for Windows. The chi-square test, Student’s *t*-test, or nonparametric test was used to compare the baseline characteristiics (mean ± SD or medians) at baseline; Student’s *t*-test was used to analyze the lipoprotein profiles at any time point of the two groups as well as for self-comparison. Repeated measures analysis of covariance (REANCOVA) was used to analyze the lipoprotein profiles, including TG, TC, LDL-C, and HDL-C levels. ANCOVA was used to analyze the LDL-C levels in the TG > 2.3 mmol/L subgroup, and the chi-square test was used to compare the changes in safety profiles. For all outcomes, a *P*-value of 0.05 was considered to indicate statistical significance, and all tests were two-sided.

## CONCLUSIONS

This clinical strudy provides evidence that combination therapy with the use of Zhibitai and low dose of Atorvastatin is safe, effective, and well-tolerated in reducing LDL-C and TG in patients with CHD or at high risk of CHD. The study suggests that the combination of the use of Zhibitai and low-dose Atorvastatin is potentially more efficacious in reducing LDL-C and TG and has fewer adverse events resulting in better patients’ compliance compared with high dose of Atorvastatin alone.

## SUPPLEMENTARY MATERIALS FIGURES AND TABLES



## Abbreviations

CHD: coronary heart disease
ASCVD: atherosclerotic cardiovascular disease
UA: unstable angina
AMI: acute myocardial infarction
TC: total cholesterol
TG: triglyceride
LDL-C: low-density lipoprotein cholesterol
HDL-C: high-density lipoprotein cholesterol
ALT: alanine transaminase
AST: aspartate transaminase
BUN: blood urea nitrogen
CK: creatine kinase
NE: nephritic syndrome
NYHA: the New York Heart Association
BMI: body mass index
SBP: systolic blood pressure
DBP: diastolic blood pressure
HMG-CoA: hydroxymethylglutaryl coenzyme A
PCSK9: protein convertase subtilisin/kexin type 9
SREBP-2: sterol regulatory element-binding protein-2
REANCOVA: Repeated measures analysis of covariance
