# Supplementary material for: Efficacy and safety of Zhibitai in combination with atorvastatin for lipid lowering in patients with coronary heart disease
Source: Oncotarget. 2017 Jun 1;9(10):9489–97. doi: 10.18632/oncotarget.18329 (PMC5823641; doi:10.18632/oncotarget.18329)
Supplement: Supplementary file 1 [file oncotarget-09-9489-s001.pdf]

Efficacy and safety of Zhibitai in combination with atorvastatin for lipid lowering in patients with coronary heart disease

SUPPLEMENTARY MATERIALS

Supplementary Table 1: Occurrence of abnormal clinical laboratory tests in patients with TG>203.72mg/dL [no. (%)]

Group / Index	Zhibitai-Atorvastatin Group		Monotherapy Group	
	Week 4	Week 8	Week 4	Week 8
ALT(abnormal)	7(4.40)	6(3.73) [a]	13(8.97)	23(15.75)
AST(abnormal)	6(3.73)	4(2.44) [b]	9(6.21)	20(13.42)
BUN(abnormal)	4(2.86)	6(4.11)	4(2.70)	8(5.41)
Creatine(abnormal)	3(2.13)	5(3.40)	0(0)	4(2.63)
CK(abnormal)	2(1.80)	3(2.73)	2(2.22)	2(2.20)

Compared with Monotherapy Group at week 8, $^{a}P < 0.001$, $^{b}P < 0.001$. ALT: Alanine transaminase (the definition of ALT abnormal was >3 times the upper limit of normal range); AST: aspartate transaminase（the definition of ALT abnormal was >3 times the upper limit of normal range)； BUN: blood urea nitrogen (the definition of BUN abnormal was > the upper limit of normal range)；CK: creatine kinase (the definition of ALT abnormal was >10times the upper limit of normal range)

Supplementary Table 2: Occurrence of abnormal clinical laboratory tests in patients with LDL-C>131.48mg/dL [no. (%)]

Group / Index	Zhibitai-Atorvastatin Group		Monotherapy Group	
	Week 4	Week 8	Week 4	Week 8
ALT(abnormal)	6(4.14)	4(2.65) [a]	15(0.34)	24(16.90)
AST(abnormal)	5(3.42)	4(2.63) [b]	11(7.69)	20(16.90)
BUN(abnormal)	7(5.88)	6(4.69)	6(4.14)	13(9.15)
Creatine(abnormal)	5(4.07)	4(3.08)	1(0.68)	7(4.83)
CK(abnormal)	5(4.81)	6(5.94)	3(3.26)	4(4.60)

Compared with Monotherapy Group at week 8, $^{a}P < 0.001$, $^{b}P < 0.001$. ALT: Alanine transaminase (the definition of ALT abnormal was >3 times the upper limit of normal range); AST: aspartate transaminase (the definition of ALT abnormal was >3 times the upper limit of normal range); BUN: blood urea nitrogen (the definition of BUN was>the upper limit of normal range)

Supplementary Table 3: Baseline characteristics of the patients (PPS)

Characteristics	Zhibitai-Atorvastatin Group (n = 350)	Monotherapy Group (n = 346)
Age, years	58.30±9.82	57.25±9.81
Sex (male/female), no.	155/195	141/205
Height, cm	164.81±7.74	165.80±8.07
Weight, kg	66.89±10.51	67.73±10.54
BMI, kg/cm^2	24.57±3.02	24.55±2.86
SBP, mmHg	135.25±17.12	134.70±16.98
DBP, mmHg	82.60±11.18	81.42±10.49
Smokers, no. (%)	129(36.86)	130(37.57)
Patients with CHD or at high risk of CHD, no. (%)	233(66.01)	241(67.51)

BMI: body mass index, SBP: systolic blood pressure, DBP: diastolic blood pressure

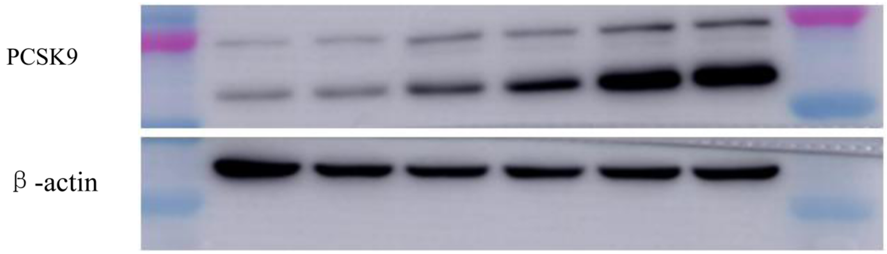

Statin(umol/l)	-	-	0.1	1	10	20
DMSO 1/1000	+	+	+	+	+	+

Supplementary Figure 1: Effects of statin on expression of PCSK9.

Effects of statin on expression of PCSK9. Human hepatoma HepG2 cells were growing to sub-confluents state and then were starved for 12 hours. These starving cells were treated with different dosage of atorvastatin. Expression of PCSK9 was detected after 24 hours by Western blot analysis.

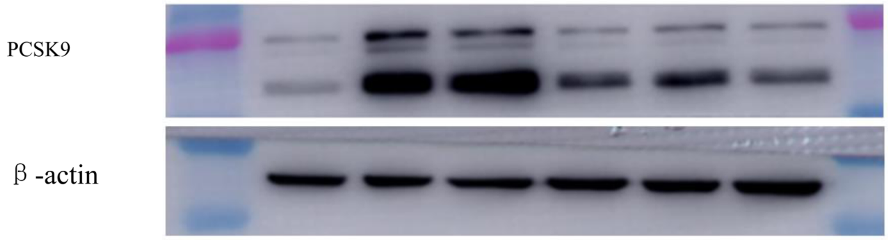

Zhibitai(ug/ml)	-	100	50	10	1	-
DMSO1/1000	-	+	+	+	+	+

Supplementary Figure 2：Effects of Zhibitai on expression of PCSK9.

Effects of Zhibitai on expression of PCSK9. Human hepatoma HepG2 cells were growing to sub-confluents state and then were starved for 12 hours. These starving cells were treated with different dosage of Zhibitai. Expression of PCSK9 was detected after 24 hours by Western blot analysis.

LXR

β -actin

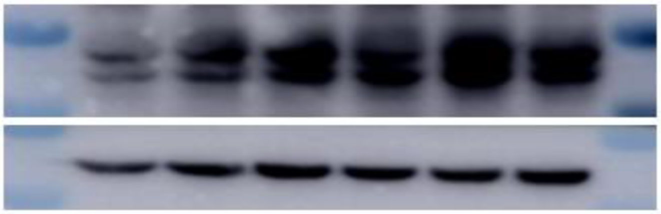

Zhibitai(ug/ml)	-	1	10	50	100	-
DMSO1/1000	+	+	+	+	+	-

Supplementary Figure 3：Effects of Zhibitai on expression of LXR.

Effects of Zhibitai on expression of LXR. Human hepatoma HepG2 cells were growing to sub-confluents state and then were starved for 12 hours. These starving cells were treated with different dosage of Zhibitai. Expression of LXR was detected after 24 hours by Western blot analysis.
